# Molecular genetics of familial nystagmus complicated with cataract and iris anomalies

**Published:** 2011-10-05

**Authors:** Naihong Yan, Yongwang Zhao, Yun Wang, Airui Xie, Haitao Huang, Wenhan Yu, Xuyang Liu, Su-ping Cai

**Affiliations:** 1 Ophthalmic Laboratories & Department of Ophthalmology, West China Hospital, Sichuan University, Chengdu, P.R. China; 2 Department of Ophthalmology, the Affiliated Hospital of Vocational Technology Institute of Yongzhou, Hunan Province, Yongzhou, P.R. China

## Abstract

**Purpose:**

Familial nystagmus complicated with cataract and iris anomalies are genetically heterogeneous, and the pathophysiological mechanisms remain unclear. It is anticipated that mutations in the paired box 6 (*PAX6*) gene play a major role in pathogenesis of malformations in anterior segment of the eye. In this study, we analyzed *PAX6* in a Chinese pedigree of nystagmus, cataract and iris anomalies. This study will provide insights into the genetic basis of this disease.

**Methods:**

Complete ophthalmologic examinations were performed on four patients (excluding one dead patient) and four unaffected individuals in this four-generation family. All coding exons of *PAX6* were amplified by polymerase chain reaction (PCR), sequenced and compared with reference database. The variations detected were evaluated in available family members as well as 110 normal controls. Possible changes in structure and function of the protein induced by amino acid variance were predicted by bioinformatics analysis.

**Results:**

Nystagmus, cataract or iris anomalies were found in all patients of this family, but the severity was different among these patients. A novel missense mutation in *PAX6* was identified in all affected individuals but not in asymptomatic members and 110 normal controls. This mutation causes an amino acid substitution of proline to glutamine at position 118 (p.P118Q) of the paired domain of the PAX6 protein. Such a change may cause structural and functional changes of the protein based on bioinformatics analysis.

**Conclusions:**

This study added a novel mutation to the existing spectrum of *PAX6* mutations, suggesting that a mutation in *PAX6* correlated with anterior segment disorders observed in this family.

## Introduction

The anterior segment is the front third of the eye that includes the structures in front of the vitreous humor: the cornea, iris, ciliary body, and lens. Human anterior segment anomalies are generally caused by disturbances early in the development of the eye and have various hereditary patterns [1,2]. Anterior segment anomalies are complex, continuous spectrum of disorders, including iris anomalies, cataract and nystagmus. Previous studies showed that a variety of anterior segment anomalies may be associated with paired box 6 (*PAX6*) gene mutations [3-5].

*PAX6* (OMIM 607108, GenBank M93650), a member of the paired box gene family, encodes a transcriptional regulator involved in oculogenesis and other developmental processes [6,7]. The human *PAX6* gene located on chromosome 11p13, with its role in oculogenesis first demonstrated as deleted or mutated *PAX6* was found in patients with iris anomalies [8,9]. *PAX6* spans 22 kb in size and consists of 14 exons and 13 introns [10]. Mutations in *PAX6* have been reported in a growing number of patients with iris anomalies and other ocular malformations [11,12]. Currently there are around 700 mutations identified and reported in the PAX6 mutation database [13].

In this study, mutation analysis of *PAX6* was performed in a large Chinese family with nystagmus, cataract, and iris anomalies.

## Methods

### Family recruitment

**Figure 1:**
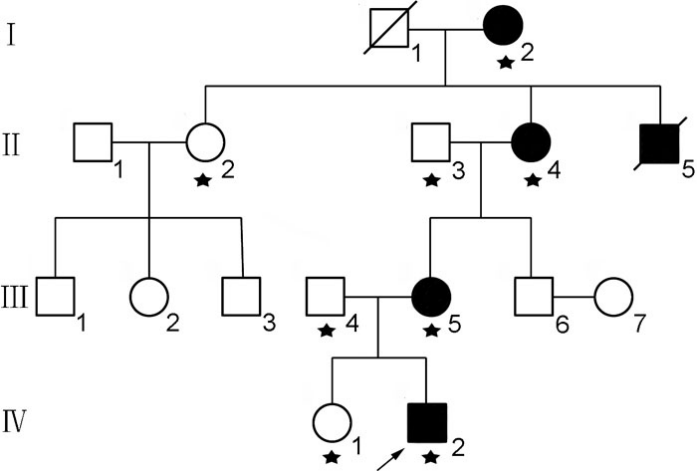
Pedigree of the Chinese family with nystagmus, cataract, and iris anomalies. Filled squares and circles are affected males and females, respectively. Arrowhead indicates proband. The asterisk indicates family members included this study.

A four-generation Chinese family with nystagmus, cataract, and iris anomalies was recruited from the clinic of Department of Ophthalmology, the Affiliated Hospital of Vocational Technology Institute of Yongzhou (Figure 1). All individuals in the control group were healthy and with no history of familial inherited ocular diseases. This study was approved by West China Hospital, Sichuan University Institute Review Board, and informed consent conforming to the tenets of the Declaration of Helsinki was obtained from all participants.

### Clinical examination

Complete physical examination and detailed ophthalmological examination were conducted by an experienced ophthalmologist on the affected and unaffected individuals, including Snellen best-corrected visual acuity test, slit-lamp microscopy examination, intraocular pressure (IOP) measurement, B-ultrasonic scan, and fundus examination.

### Mutation screening and sequence analysis

Genomic DNA was extracted from 200 μl venous blood using a Qiamp Blood Kit (Qiagen, Hilden, Germany). All the procedures were performed according to manufacturer’s protocol. DNA integrity was evaluated by 1% agarose gel electrophoresis.

The coding exons 4–13 of *PAX6* with intronic flanking sequences were amplified by PCR using previously published primers [2,12] (Table 1). The primers were synthesized by Invitrogen Company (Carlsbad, CA). PCRs were performed in a MyCycler thermocycler (Bio-Rad, Hercules, CA) using the following program: initial denaturation at 95 °C for 2 min followed by 35 cycles of 94 °C for 10 s, 51 °C-56 °C for 30 s, and 72 °C for 1 min, and final extension at 72 °C for 5 min.

**Table 1:** Primers used in polymerase chain reaction for amplification of *PAX6*.

**Exon**	**Primer direction **	**Sequence (5′→3′)**	**Annealing temperature (°C)**	**Product size (bp)**
4	Forward:	AAGGGTAGATTTTGTATGCAC	54	276
	Reverse:	GAAGTCCCAGAAAGACCAGA		
5	Forward:	CCTCTTCACTCTGCTCTCTT	54	257
	Reverse:	ATGAAGAGAGGGCGTTGAGA		
5a and 6	Forward:	TGAAAGTATCATCATATTTGTAG	54	515
	Reverse:	AGGAGAGAGCATTGGGCTTA		
7	Forward:	CAGGAGACACTACCATTTGG	56	265
	Reverse:	GACAGGCAAAGGGATGCAC		
8	Forward:	GGGAATGTTTTGGTGAGGCT	54	346
	Reverse:	TCTTTGTACTGAAGATGTGGC		
9	Forward:	GTAGTTCTGGCACAATATGG	51	329
	Reverse:	GCACTGTGTCTACGTCGAG		
10 and 11	Forward:	CTCGACGTAGACACAGTGC	54	437
	Reverse:	TTATGCAGGCCACCACCAGC		
12	Forward:	GCTGTGTGATGTGTTCCTCA	54	245
	Reverse:	AAGAGAGATCGCCTCTGTGC		

﻿PCR products were directly sequenced using an ABI 377XL automated DNA sequencer (Applied Biosystems, Foster City, CA). Sequence data were compared pair-wise with the published *PAX6* sequence. Mutation was named according to the nomenclature recommended by the Human Genomic Variation Society (HGVS).

### Bioinformatics analysis

The Clustalw tool was used to align the protein sequences among eight different species. The possible functional impact of an amino acid change was predicted by Polyphen and SIFT. The isoelectric point (pI) and molecular weight of the wild type and mutant protein were analyzed by the Compute pI/Mw tool. The mutation region in 3D structure of PAX6 protein was analyzed by the Cn3D tool.

## Results

### Clinical findings of the examined family

Four patients in four successive generations of this family were found to have a similar congenital ocular disorder. The segregation of the ocular anomaly is consistent with an autosomal dominant inheritance.

**Figure 2:**
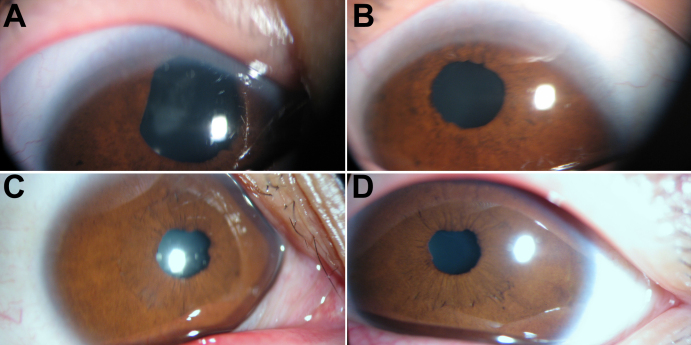
Anterior segment of proband (IV:2) and the proband’s mother (III:5). **A** and **B** show iris anomalies, and nasally and superiorly displaced pupil and cataract in both eyes of proband (IV:2). **C** and **D** show mild nasally displaced pupil and cataract of the proband’s mother (III:5).

The proband (patient IV:2, a seven-year-old boy, Figure 1) presented with nystagmus, cataract, and iris anomalies (corectopia and coloboma of iris). In addition, this patient also presented with mental retardation and muscle spasms. His corrected visual acuity was 0.1 OD and 0.1 OS. Intraocular pressure (IOP) was 17 mmHg in the right eye and 16.8 mmHg in the left eye. He was noted to have nystagmus after birth. Clinically, he showed rapid jerk nystagmus with poorly developed fixation ability (Figure 2A,B). B-scanning revealed abnormally short axial lengths and deep anterior chambers (data not shown). Fundus details could not be seen, but retinas were attached per ultrasound.

The proband’s mother (Patient III:5, thirty-four-year-old, Figure 1) was diagnosed with nystagmus, cataract, and mild iris defects (irregular pupils and mild corectopia; Figure 2C,D). Her visual acuity was 0.1 OD and 0.1 OS, and IOP was 10 mmHg OD and 13 mmHg OS. Fundus details could not be seen.

The proband’s grandmother (Patient II:4, fifty-three-year-old, Figure 1) had mental illness. She became blind ten years ago and had nystagmus, cataract and anomalies of iris (data not shown). The proband’s great-grandmother (Patient I:2, eighty-five-year-old, Figure 1) had various illness and was bed-ridden and couldn’t be examined by slit-lamp microscopy. She was blind for more than 30 years and had nystagmus, cataract, and iris anomalies (She was examined with direct ophthalmoscope and flashlight). Patient II:5 was deceased in his thirties by accident. His elder sister recalled that his vision was very poor and had nystagmus at his early age.

Ocular abnormalities were not found in four unaffected members examined in this family.

### *PAX6* mutation identification and analysis

**Figure 3:**
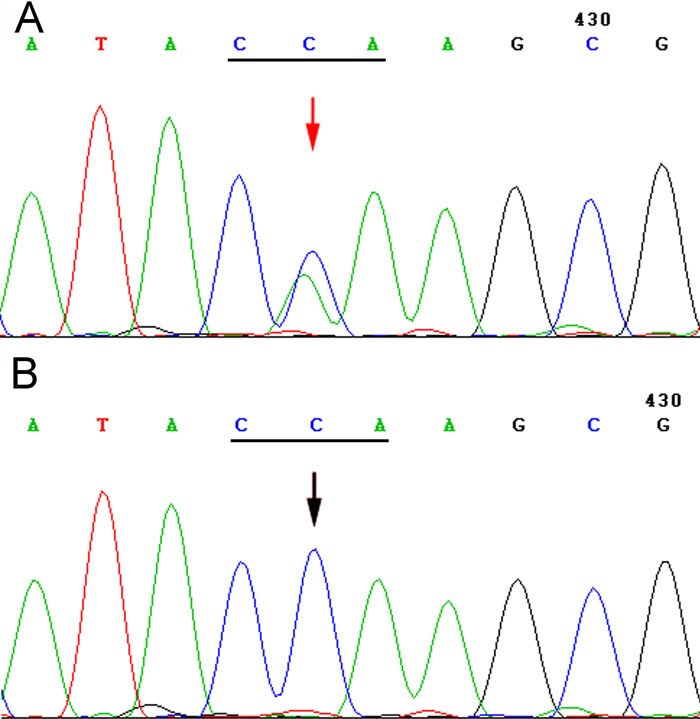
Sequencing results of the *PAX6* gene. **A**: a heterozygous C to A transversion at codon 118 in one patient from the family (arrowhead). **B**: Wild type sequence from an unaffected member.

A novel heterozygous mutation, c.353 C>A, at codon 118 (CCA to CAA) of exon 6 in *PAX6* was identified. This heterozygous mutation was present in all affected individuals whereas none of the unaffected family members and 110 normal control subjects examined carried the mutation (Figure 3). The affected individuals were heterozygous alleles with mutant and wild-type alleles. The unaffected members carried wild-type alleles.

### Bioinformatics analysis

**Figure 4:**
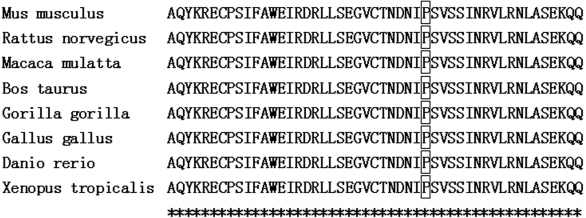
The mutation involved a highly conserved residue. The proline at position 118 is highly conserved for PAX6, which was demonstrated by analysis of orthologs from eight different species.

**Figure 5:**
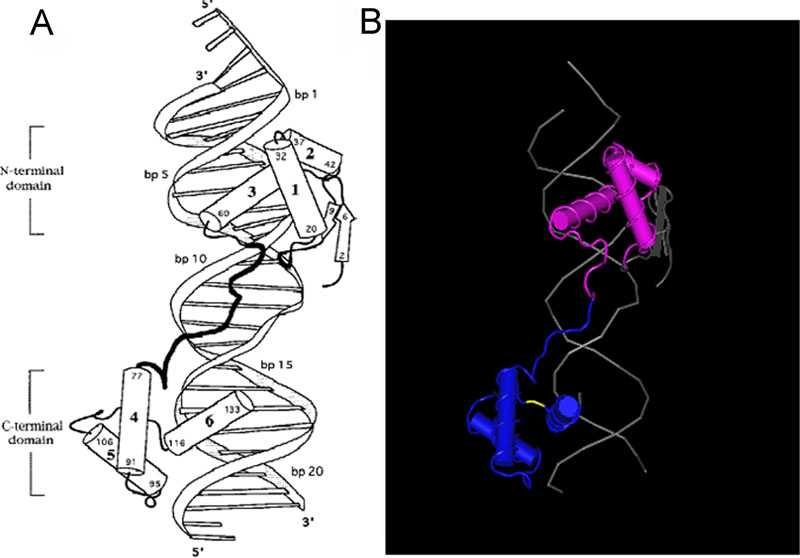
The paired domain of PAX6. **A**: Sketch of the PAX6 paired domain–DNA complex. Cylinders represent α helices; arrows represent β strands. Helices 1–6 are labeled; residue numbers indicate termini of the corresponding secondary structure elements. **B**: Cn3D display for the paired domain of PAX6. The yellow segment represents the mutation region (COOH-terminal region in PAX6 paired domain).

PAX6 has two DNA-binding domains, a paired domain (PD) and a homeodomain (HD) and this mutation is in a paired domain. PAX6 with the c.353 C>A mutation would result in replacement of proline by glutamine, leading to a change of a hydrophobic amino acid to a hydrophilic amino acid. Proline in this position was found highly conserved for PAX6 by analyzing orthologs from eight different species using Clustalw tool on line (Figure 4). The p.P118Q mutation was predicted to be “probably damaging” by Polyphen with a score of 2.736, and “affect protein function” by SIFT with a score of 0.00. The theoretical pI of mutant PAX6 was 9.45 and no change compared with wild type PAX6. The Mw of the mutant PAX6 was slightly increased to 46714.39 Da from the wild type PAX6 of 46683.37 Da. Cn3D tool analysis also showed that p.P118Q mutation was located in the COOH-terminal region of PAX6 paired domain (Figure 5) [14].

## Discussion

All patients of this Chinese pedigree had different degree of anterior segment anomalies since childhood. A 7-year-old patient also presented with mental retardation and muscle spasms. The disease in this family showed autosomal dominant inheritance and the main symptoms included nystagmus, cataract, and iris anomalies. The pathophysiological mechanisms underlying this disease remain unclear. Many studies showed that *PAX6* is important for development of the eye and central nervous tissues. Dysfunction of *PAX6* causes defects in eye development from *Drosophila* to human, as *PAX6* mutations are known to cause eyeless in *Drosophila*, small-eye phenotype in the mouse, and iris anomalies and Peters' anomaly in humans [3,15,16]. To date, genetic analysis has detected numerous mutations of PAX6 in patients with iris anomalies [13]. In this study, a novel heterozygous mutation (c.353 C>A, p.P118Q) in *PAX6* was identified in a Chinese pedigree. This mutation was identified in all affected patients (I:2, II:4, III:5, and IV:2), but not in unaffected family menbers and normal control subjects. This mutation was predicted to be deleterious by both Polyphen and SIFT with consistent results. Therefore, it was considered that this variation appears to be a causative mutation of the disease in this family.

PAX6 may regulate the expression of other genes during embryogenesis as a transcription factor [4,10]. *PAX6* expresses two isoforms by alternative splicing, PAX6 (−5a; 422 amino acids) and PAX6 (+5a; 436 amino acids) [14,17]. The paired domain (PD) and homeodomain (HD) are two DNA-binding domains of PAX6 and the paired domain consists of 128 amino acids and contains two globular subdomains (NH_2_-subdomain and COOH-subdomain) linked by an extended polypeptide chain [18,19]. This novel p.P118Q mutation was in the COOH-terminal region of the paired domain. The p.P118Q mutation turned a hydrophobic amino acid (proline) into a hydrophilic amino acid (glutamine), and since it is located in the hydrophobic core of the COOH-subdomain, it may disrupt the folding and stability of the whole protein (Figure 5). The proline at codon 118 is highly conserved in PAX6 paired-domains of all species, including *Mus musculus*, *Rattus norvegicus*, *Macaca mulatta*, *Bos taurus*, *Gorilla gorilla*, *Gallus gallus*, *Danio rerio*, and *Xenopus tropicalis* (Figure 4). Such a high degree of conservation argues for a functional importance of the relevant amino acid residue.

A similar mutation, p.P118R (c.353 C>G), of *PAX6* was previously found in a Japanese family [5]. This family showed a variety of congenital ocular disorders, including aniridia, congenital nystagmus, minimal displaced pupil, and foveal hypoplasia. The proline to glutamine (p.P118Q) substitution in the COOH-terminal part of the paired domain reported here has a phenotypic outcome different from that seen when the same residue is substituted with arginine (p.P118R). The Human *PAX6* Allelic Variant Database showed that at the same codon more than 1 variant missense base-pair mutation leading to production of different amino acids is found. This illustrates how different missense mutations affecting the same codon of *PAX6* may result in distinctly different phenotypes [20].

The affected individuals in this family showed congenital ocular disorders, including nystagmus, cataract, and iris anomalies. Besides these ocular symptoms, the proband (IV:2) also had mental retardation and skeletal muscle spasms. His grandmother (II:4) was diagnosed as a schizophrenic disorder in her early 30s. *PAX6* expresses in the developing neural tissues of the brain, such as cerebellum, and plays a role in the development of neural system and the eye [5,21]. Some *PAX6* mutations may play a critical role in controlling the migration and differentiation of specific neuronal progenitor cells in the brain [22]. Graziano et al. [23] reported a de novo nonsense mutation S119R (serine to arginine) of *PAX6* in a patient with aniridia, ataxia, and mental retardation. This mutation may be responsible for aniridia, ptosis and cognitive dysfunction. Davis et al. [24] analyzed of a patient with aniridia, autism, and mental retardation and identified a 1.3 Mb deletion of *PAX6*. Other findings also provided evidence that the role of *PAX 6* in brain anomalies associated with iris anomalies [25,26].

In summary, this study added a novel missense mutation to the existing spectrum of *PAX6* mutations in a Chinese family with nystagmus, cataract and iris anomalies. This study provides further evidence that haploinsufficiency of the *PAX6* gene causes defects in eye development and it also adds more evidences for genetic diagnosis of these inherited disorders.

## References

[1] Gould DB, John SW (2002). Anterior segment dysgenesis and the developmental glaucomas are complex traits.. Hum Mol Genet.

[2] Alward WLM (2000). Axenfeld-Rieger syndrome in the age of molecular genetics* 1.. Am J Ophthalmol.

[3] Hanson IM, Fletcher JM, Jordan T, Brown A, Taylor D, Adams RJ, Punnett HH, van Heyningen V (1994). Mutations at the PAX6 locus are found in heterogeneous anterior segment malformations including Peters' anomaly.. Nat Genet.

[4] Chi N, Epstein JA (2002). Getting your Pax straight: Pax proteins in development and disease.. Trends Genet.

[5] Sonoda S, Isashiki Y, Tabata Y, Kimura K, Kakiuchi T, Ohba N (2000). A novel PAX6 gene mutation (P118R) in a family with congenital nystagmus associated with a variant form of aniridia.. Graefes Arch Clin Exp Ophthalmol.

[6] Collinson JM, Quinn JC, Hill RE, West JD (2003). The roles of Pax6 in the cornea, retina, and olfactory epithelium of the developing mouse embryo.. Dev Biol.

[7] Grindley JC, Davidson DR, Hill RE (1995). The role of Pax-6 in eye and nasal development.. Development.

[8] Ton CCT, Hirvonen H, Miwa H, Weil MM, Monaghan P, Jordan T, Van Heyningen V, Hastie ND, Meijers-Heijboer H, Drechsler M (1991). Positional cloning and characterization of a paired box-and homeobox-containing gene from the aniridia region.. Cell.

[9] Jordan T, Hanson I, Zaletayev D, Hodgson S, Prosser J, Seawright A, Hastie N, van Heyningen V (1992). The human PAX6 gene is mutated in two patients with aniridia.. Nat Genet.

[10] Epstein JA, Glaser T, Cai J, Jepeal L, Walton DS, Maas RL (1994). Two independent and interactive DNA-binding subdomains of the Pax6 paired domain are regulated by alternative splicing.. Genes Dev.

[11] Prosser J, van Heyningen V (1998). PAX6 mutations reviewed.. Hum Mutat.

[12] Brown A, McKie M, van Heyningen V, Prosser J (1998). The Human PAX6 Mutation Database.. Nucleic Acids Res.

[13] Lalonde E, Albrecht S, Ha KC, Jacob K, Bolduc N, Polychronakos C, Dechelotte P, Majewski J, Jabado N (2010). Unexpected allelic heterogeneity and spectrum of mutations in Fowler syndrome revealed by next-generation exome sequencing.. Hum Mutat.

[14] Xu W, Rould MA, Jun S, Desplan C, Pabo CO (1995). Crystal structure of a paired domain-DNA complex at 2.5 resolution reveals structural basis for Pax developmental mutations.. Cell.

[15] Hill RE, Favor J, Hogan BL, Ton CC, Saunders GF, Hanson IM, Prosser J, Jordan T, Hastie ND, van Heyningen V (1991). Mouse small eye results from mutations in a paired-like homeobox-containing gene.. Nature.

[16] Quiring R, Walldorf U, Kloter U, Gehring WJ (1994). Homology of the eyeless gene of Drosophila to the Small eye gene in mice and Aniridia in humans.. Science.

[17] Czerny T, Schaffner G, Busslinger M (1993). DNA sequence recognition by Pax proteins: bipartite structure of the paired domain and its binding site.. Genes Dev.

[18] Glaser T, Walton DS, Maas RL (1992). Genomic structure, evolutionary conservation and aniridia mutations in the human PAX6 gene.. Nat Genet.

[19] Xu HE, Rould MA, Xu W, Epstein JA, Maas RL, Pabo CO (1999). Crystal structure of the human Pax6 paired domain¨CDNA complex reveals specific roles for the linker region and C-terminal subdomain in DNA binding.. Genes Dev.

[20] Bredrup C, Knappskog PM, Rodahl E, Boman H (2008). Clinical manifestation of a novel PAX6 mutation Arg128Pro.. Arch Ophthalmol.

[21] Callaerts P, Halder G, Gehring WJ (1997). PAX-6 in development and evolution.. Annu Rev Neurosci.

[22] Glaser T, Jepeal L, Edwards JG, Young SR, Favor J, Maas RL (1994). PAX6 gene dosage effect in a family with congenital cataracts, aniridia, anophthalmia and central nervous system defects.. Nat Genet.

[23] Graziano C, D'Elia AV, Mazzanti L, Moscano F, Guidelli Guidi S, Scarano E, Turchetti D, Franzoni E, Romeo G, Damante G (2007). A de novo nonsense mutation of PAX6 gene in a patient with aniridia, ataxia, and mental retardation.. Am J Med Genet A.

[24] Davis LK, Meyer KJ, Rudd DS, Librant AL, Epping EA, Sheffield VC, Wassink TH (2008). Pax6 3′ deletion results in aniridia, autism and mental retardation.. Hum Genet.

[25] Malandrini A, Mari F, Palmeri S, Gambelli S, Berti G, Bruttini M, Bardelli AM, Williamson K, van Heyningen V, Renieri A (2001). PAX6 mutation in a family with aniridia, congenital ptosis, and mental retardation.. Clin Genet.

[26] Abouzeid H, Youssef MA, ElShakankiri N, Hauser P, Munier FL, Schorderet DF (2009). PAX6 aniridia and interhemispheric brain anomalies.. Mol Vis.

